# Supplementary material for: Bony Fish Arachidonic Acid 15-Lipoxygenases Exhibit Different Catalytic Properties than Their Mammalian Orthologs, Suggesting Functional Enzyme Evolution during Vertebrate Development
Source: Int J Mol Sci. 2023 Sep 15;24(18):14154. doi: 10.3390/ijms241814154 (PMC10531496; doi:10.3390/ijms241814154)
Supplement: Supplementary file 1 [file ijms-24-14154-s001.zip › ijms-2580525-supplementary.pdf]

**Supplement for the paper**

# Bony Fish Arachidonic Acid 15-Lipoxygenases Exhibit Different Catalytic Properties than Their Mammalian Orthologs, Suggesting Functional Enzyme Evolution during Vertebrate Development

**Sophie Roigas [1], Kumar R. Kakularam [1], Michael Rothe [2], Dagmar Heydeck [1], Polamarasetty Aparoy [3] and Hartmut Kuhn [1,*]**

[1] Department of Biochemistry, Charité-Universitätsmedizin Berlin, Corporate Member of Freie Universität Berlin and Humboldt Universität zu Berlin, Charitéplatz 1, 10117 Berlin, Germany; sophie.roigas@charite.de (S.R.); kumar.1416@gmail.com (K.R.K.); dagmar.heydeck@charite.de (D.H.)

[2] Lipidomix GmbH, Robert-Rössle-Straße 10, 13125 Berlin, Germany; michael.rothe@lipidomix.de

[3] Department of Humanities and Sciences, Indian Institute of Petroleum and Energy, Visakhapatnam 530003, India; aparoy@gmail.com

* Correspondence: hartmut.kuehn@charite.de; Tel.:+49-30-450528040

**Keywords:** eicosanoids; lipoxygenase; enzyme evolution; vertebrates; mammals

## 1. Methodological supplement

### 1.1 cDNA subcloning and enzyme expression of putative bony fish ALOX15 orthologs

For further subcloning from the initial puC57 synthesis vector into pET28b(+) (Novagen/Merck, Darmstadt, Germany) or pFastBac HT expression vectors, a SalI restriction site was introduced immediately upstream of the start codon and a HindIII restriction site was generated immediately downstream of the stop codon. The sequence was optimized for bacterial expression by silent mutations. Finally, the recombinant plasmid was digested with SalI and HindIII to check for the ALOX15 insert and a positive clone involving the ALOX insert was sequenced (Eurofins Genomics Germany GmbH, Ebersberg, Germany). The expression of the enzymes as N-terminal hexa-His-tag fusion proteins was performed as as follows: *E. coli* Rosetta2 (DE3)-pLysS cells were transformed with the recombinant plasmid pET28b and grown on an agar plate containing kanamycin and chloramphenicol. An isolated clone was seleted and grown under shaking in a glucose free MSM with added trace elements and glucoamylase. Protein expression was induced by addition of IPTG. After 18 h cells were harvested, homogenized by sonication and the cell free supernatant was used as enzyme source. Since some putative bony fish ALOX15 orthologs are not well expressed in E. coli we expressed these enzyme in insect cells. For this purpose, the coding sequence was subcloned into the pFastBac HT vector and the bacmid as well as the recombinant baculovirus were generated according to the manufacturer’s instructions (Bac-to-Bac® Baculovirus Expression System, Invitrogen Life Technologies/Thermo Fisher, Schwerte, Germany). The cells were infected with virus and incubated on a shaking platform until about 30% dead cells were recorded (on average after 72 h, tested by trypane blue exclusion). Next, cells were harvested and lysed by sonication and the cell free lysate supernatant was used as enzyme source.

### 1.2 Mutagenesis studies

Plasmid DNA containing the coding sequence of the bony fish ALOX15 isoforms was incubated with specific primer pairs containing the required changes in their nucleotide sequence to achieve the intended amino acid exchanges and Pfu Ultra II Hot Start 2 × PCR Master Mix (Agilent Technologies, Waldbronn, Germany). After the PCR protocol (18 cycles), parent DNA was digested using DpnI. E. coli XL-1 Blue competent cells (Agilent Technologies Inc., Santa Clara, USA) were transformed with the mutated plasmid and after replication the plasmid DNA of 1 clone was sequenced (Eurofins Genomics Germany GmbH, Ebersberg, Germany).

### 1.3 In vitro activity assays and RP-HPLC analysis of the reaction products

Different amounts of the cell free supernatants were incubated with 100 µM arachidonic acid and the reduced reaction products were analyzed by RP-HPLC on a Shimadzu instrument connected with a Hewlett Packard diode array detector 1040 A. Metabolites were separated on a Nucleodur $C_{18}$ Gravity column (Macherey-Nagel, Düren, Germany; 250 × 4 mm, 5 µm particle size) coupled with a corresponding guard column (8 × 4 mm, 5 µm particle size). A solvent system consisting of acetonitrile:water:acetic acid (70 : 30 : 0.1, by vol) was used at a flow rate of 1 $mL \cdot min^{-1}$. For more detailed analysis of the products structures the conjugated dienes were prepared by RP-HPLC and further analyzed by normal-phase HPLC (NP-HPLC) and/or chiral-phase HPLC (CP-HPLC). Normal-phase HPLC was perfomed using the solvent system n-hexane/2-propanol/acetic acid (100/2/0.1, by volume) on a Nucleosil 100-5 column (250 × 4.6 mm, 5 µm particle size). 12-HETE enantiomers were resolved on a Chiralpak AD-H column (Daicel Corp., Osaka, Japan) with a solvent system consisting of n-hexane/methanol/ ethanol/acetic acid (96 : 3 : 1 : 0.1, by vol; 1 mL/min).

## 2. Supplemental experimental data

**Table S1. Degree of amino acid identity for the different putative bony fish ALOX15 orthologs.** Dual amino acid sequence alignments were carried out with the Emboss Needle (https://www.ebi.ac.uk/Tools/psa/emboss_needle/ accessed on 25 April 2019) program and the degree of amino acid identity (in %) are given as measure for the evolutionary relatedness.

| Species | Amino acid identity (%) | | | | |
|---|---|---|---|---|---|
| | *D. rerio* | *N. furzeri* | *P. nyererei* | *S. formosus* | *H. burtoni* |
| *D. rerio* | 100 | 75 | 75 | 75 | 71 |
| *N. furzeri* | 75 | 100 | 86 | 74 | 81 |
| *P. nyererei* | 75 | 86 | 100 | 75 | 93 |
| *S. formosus* | 75 | 74 | 75 | 100 | 71 |
| *H. burtoni* | 71 | 81 | 93 | 71 | 100 |

**Table S2. Degree of amino acid conservation between the putative bony fish ALOX15 orthologs and human ALOX-isoforms.** Dual amino acid sequence alignments were carried out with the Emboss Needle (https://www.ebi.ac.uk/Tools/psa/emboss_needle/ accessed on 25 April 2019) program and the degree of amino acid identity (in %) are given as suitable measure for the evolutionary relatedness.

| Species | Amino acid identity to different human ALOX isoforms (%) | | | | | |
|---|---|---|---|---|---|---|
| | ALOX15 | ALOX15B | ALOX12 | ALOX12B | ALOXE3 | ALOX5 |
| *D. rerio* | 44 | 43 | 46 | 41 | 40 | 45 |
| *N. furzeri* | 44 | 43 | 45 | 41 | 41 | 48 |
| *P. nyererei* | 44 | 43 | 47 | 41 | 41 | 47 |
| *S. formosus* | 44 | 43 | 45 | 42 | 41 | 47 |

**Table S3. Degree of amino acid conservation between the different putative bony fish ALOX15 orthologs and mouse Alox-isoforms.** Dual amino acid sequence alignments were carried out with the Emboss Needle (https://www.ebi.ac.uk/Tools/psa/emboss_needle/ accessed on 25 April 2019) program and the degree of amino acid identity (in %) are given as measure for the evolutionary relatedness.

| Species | Amino acid identity to different mouse ALOX- isoforms (%) | | | | | | |
|---|---|---|---|---|---|---|---|
| | Alox15 | Alox15b | Alox12 | Alox12b | Aloxe3 | Alox5 | Alox12e |
| *D. rerio* | 43 | 41 | 46 | 39 | 40 | 44 | 42 |
| *N. furzeri* | 44 | 42 | 46 | 40 | 42 | 46 | 42 |
| *P. nyererei* | 44 | 42 | 48 | 40 | 43 | 46 | 44 |
| *S. formosus* | 43 | 41 | 47 | 40 | 41 | 46 | 43 |

## Table S4 Number of species evaluated in relation to the overall number of described species, and numbers of threatened species by major groups of organisms.

| | Estimated Number of described species[1] | Nmber of species evaluated by 2022 (IUCN Red List version 2022-1) | % of described species evaluated by 2022 (IUCN Red List version 2022-1) | Number of threatened species[2] by 2022 (IUCN Red List versiom 2022-1) | Estimated % threatened species in 2022 (IUCN Red List version 2022-1)[2,3,4] | | |
|---|---|---|---|---|---|---|---|
| | | | | | Lower estimate (threatened spp. as % of extant evaluated species) | Best estimate (threatened spp. as % of extant data sufficient evaluated species) | Upper stimate (threatened and DD spp. as% of extant evaluated species) |
| **VERTEBRATES** | | | | | | | |
| Mammals [5] | 6,577 | 5,969 | 91% | 1,337 | 23% | 26% | 37% |
| Birds | 11,162 | 11,162 | 100% | 1,409 | 13% | 13% | 13% |
| Reptiles | 11,690 | 10,150 | 87% | 1,845 | 18% | 21% | 33% |
| Amphibians | 8,463 | 7,316 | 86% | 2,515 | 35% | 41% | 51% |
| Fishes | 36,248 | 24,356 | 67% | 3,548 | *Insufficient coverage* | | |
| **Subtotal** | **74,140** | **58,953** | **80%** | **10,654** | | | |
| **INVERTEBRATES** | | | | | | | |
| Insects | 1,053,578 | 12,161 | 1.2% | 2,291 | *Insufficient coverage* | | |
| Molluscs | 84,528 | 9,017 | 11% | 2,384 | *Insufficient coverage* | | |
| Crustaceans[6] | 80,122 | 3,197 | 4% | 745 | *Insufficient coverage* | | |
| Corals | 5,574 | 846 | 15% | 232 | *Insufficient coverage* | | |
| Arachnids | 110,615 | 441 | 0.40% | 251 | *Insufficient coverage* | | |
| Velvet Worms | 210 | 11 | 5% | 9 | *Insufficient coverage* | | |
| Horseshoe Crabs | 4 | 4 | 100% | 2 | 50% | 100% | 100% |
| Others | 157,543 | 904 | 0.57% | 152 | *Insufficient coverage* | | |
| **Subtotal** | **1,492,174** | **26,581** | **2%** | **6,066** | | | |
| **PLANTS [7]** | | | | | | | |
| Mosses [8] | 21,925 | 282 | 1.3% | 165 | *Insufficient coverage* | | |
| Ferns and Allies [9] | 11,800 | 747 | 6% | 288 | *Insufficient coverage* | | |
| Gymnosperms | 1,113 | 1,046 | 94% | 436 | 2% | 2% | 44% |
| Flowering Plants | 369,000 | 59,222 | 16% | 23,551 | *Insufficient coverage* | | |
| Green Algae [10] | 12,382 | 16 | 0.1% | 0 | *Insufficient coverage* | | |
| Red Algae [10] | 7,480 | 58 | 0.8% | 9 | *Insufficient coverage* | | |
| **Subtotal** | **423,700** | **61,371** | **14%** | **24,449** | | | |
| **FUNGI & PROTISTS [11]** | | | | | | | |
| Lichens | 17,000 | 86 | 0.5% | 62 | *Insufficient coverage* | | |
| Mushrooms, etc. | 120,000 | 511 | 0.4% | 222 | *Insufficient coverage* | | |
| Brown Algae [10] | 4,485 | 15 | 0.3% | 6 | *Insufficient coverage* | | |
| **Subtotal** | **141,485** | **612** | **0.4%** | **290** | | | |
| **TOTAL** | **2,131,499** | **147,517** | **7%** | **41,459** | | | |

## NOTES:

**1.** The numbers of described species in Table 1a should be used with caution as these are not always be up to date for all taxonomic groups. The sources used for the figures currently shown in the table are listed below.

2. Threatened species are those listed as Critically Endangered (CR), Endangered (EN) or Vulnerable (VU).

3. Where <80% of species within a group have been evaluated, figures for % threatened species are not provided because there is insufficient coverage for these groups. It is only possible to provide reliable figures for % threatened species for those groups that are completely or almost completely evaluated (e.g., mammals, birds, amphibians and gymnosperms).

4. he percentage of threatened species can be calculated for those groups that are completely or almost completely evaluated (>80% of species evaluated), but the actual number of threatened species is often uncertain because it is not known whether Data Deficient (DD) species are actually threatened or not. Therefore, a range of percentages is provided: **lower estimate** = % threatened extant species (if all DD species are not threatened); **best estimate** = % threatened extant species (if DD species are equally threatened as data sufficient species); **upper estimate** = % threatened extant species (if all DD species are threatened). If a single figure is required for reporting purposes, the best estimate figure should be used.

5. The number of described and evaluated mammals excludes domesticated species like sheep (*Ovis aries*), goats (*Capra hircus*), Dromedary (*Camelus dromedarius*), etc.

6. Crustaceans include six classes: Branchiopoda (fairy shrimp, clam shrimp, etc.); Cephalocardia (horseshoe shrimp);Malacostraca (crabs, lobsters, shrimp, woodlice, etc.); Maxillopoda (barnacles, copepods, etc.); Ostracoda (seed shrimp) and Remipedia (remipedes)

7. The plant numbers **DO NOT** include species from the *1997 IUCN Red List of Threatened Plants* (Walter and Gillett 1998) as those assessments used the pre-1994 IUCN system of threat categories. Hence the numbers of threatened plants in Table 1b are much lower when compared to the 1997 results. When reporting on threatened plants, the results from the current web version of The IUCN Red List should be combined with the 1997 Plants Red List. Since there have been many taxonomic changes for plant species since 1997, careful comparison of the current and 1997 species lists will be needed when combining these results to avoid double-counting.

8. Mosses include the true mosses (Bryophyta), the hornworts (Anthoceratophyta), and liverworts (Marchantiophyta).

9. The ferns and allies include club mosses and spike mosses (Lycopodiopsida), quillworts (Isoetopsida), horsetails (Equisetopsida) and ferns (Marattiopsida, Polypodiopsida and Psilotopsida).

10. Seaweeds are included in the green algae (Chlorophyta, Charophyta), red algae (Rhodophyta), and brown algae (Ochrophyta).

11. Many of the decribed species in these groups are not elegible for assessment on the IUCN Red List as they are considered micro-organisms.

## Sources for Numbers of Described Species:

### Vertebrates

**Mammals** – Mammal Diversity Database. 2022. v. 1.9, released 1 April 2022. www.mammaldiversity.org. American Society of Mammalogists. Accessed 22 June 2022. The ASM Biodiversity Committee stewards the Mammal Diversity Database, an updatable and online database of mammal taxonomic and biodiversity information. Partly based on Wilson, D.E. and Reeder, D.M. (eds). 2005. Mammal Species of the World, 3rd Edition. John Hopkins University Press, Baltimore (available at https://www.departments.bucknell.edu/biology/resources/msw3/), updated using the IUCN Red List and other literature. The IUCN Red List deviates from Wilson and Reeder (2005), especially in cases where there are alternative taxonomic treatments; in such cases the Global Mammal Assessment coordinating team working with the relevant IUCN SSC Specialist Group advise on which treatment to follow. A number of differences and deviations are also based on new revisions and published papers that have appeared since the accounts in Wilson and Reeder (2005) were published. There are a number of recently described species which are currently under review and hence these are not included in the numbers cited here.

**Birds** – Handbook of the Birds of the World and BirdLife International. 2021. Handbook of the Birds of the World and BirdLife International digital checklist of the birds of the world. Version 6. Available at: http://datazone.birdlife.org/userfiles/file/Species/Taxonomy/HBW-BirdLife_Checklist_v6_Dec21.zip. Accessed: 23 June 2022

**Reptiles** – Based on the figures (as of November 2021) provided by The Reptile Database compiled by Peter Uetz and Jirí Hošek. Available at: http://www.reptile-database.org. Accessed: 23 June 2022. For current total number of species on this website, see http://reptile-database.reptarium.cz/

**Amphibians** – Frost, D.R. 2022. Amphibian Species of the World: an Online Reference. Version 6.1 (23 June 2022). Electronic Database accessible at: https://amphibiansoftheworld.amnh.org/index.php. American Museum of Natural History, New York, USA. doi.org/10.5531/db.vz.0001.

**Fishes** – Based on Frick, R. Eschmeyer, W.N. and Van der Lan, R. (eds). 2022. Eschmeyer's Catalog of Fishes: genera, species, references (http://researcharchive.calacademy.org/research/ichthyology/catalog/fishcatmain.asp). Electronic version accessed: 06 June 2022.

### Invertebrates

**Insects** – Roskov Y., Ower G., Orrell T., Nicolson D., Bailly N., Kirk P.M., Bourgoin T., DeWalt R.E., Decock W., Nieukerken E. van, Zarucchi J., Penev L., eds. (2019). Species 2000 & ITIS Catalogue of Life, 2019 Annual Checklist. Digital resource at http://www.catalogueoflife.org/annual-checklist/2019/info/totals. Species 2000: Naturalis, Leiden, the Netherlands. ISSN 2405-884X. Accessed 21 July 2021.

**Crustaceans** – Roskov Y., Ower G., Orrell T., Nicolson D., Bailly N., Kirk P.M., Bourgoin T., DeWalt R.E., Decock W., Nieukerken E. van, Zarucchi J., Penev L., eds. (2019). Species 2000 & ITIS Catalogue of Life, 2019 Annual Checklist. Digital resource at www.catalogueoflife.org/annual-checklist/2019. Species 2000: Naturalis, Leiden, the Netherlands. ISSN 2405-884X. Accessed 05 August 2021.

**Molluscs** – MolluscaBase (2022). MolluscaBase. Available at http://www.molluscabase.org. Accessed: 23 June 2022.

**Corals** – Corals fall under the phylum Cnidaria and are primarily in the class Anthozoa (orders Alcyonacea, Antipatharia, Corallimorpharia, Helioporacea, Scleractinia, although there are some in the class Hydrozoa (family Milleporidae). The number of described living species reported here are from Roskov Y., Ower G., Orrell T., Nicolson D., Bailly N., Kirk P.M., Bourgoin T., DeWalt R.E., Decock W., Nieukerken E. van, Zarucchi J., Penev L., eds. (2019). Species 2000 & ITIS Catalogue of Life, 2019 Annual Checklist. Digital resource at www.catalogueoflife.org/annual-checklist/2019. Species 2000: Naturalis, Leiden, the Netherlands. ISSN 2405-884X. Accessed: 23 June 2022

**Arachnids (spiders, scorpions, etc)** – Roskov Y., Ower G., Orrell T., Nicolson D., Bailly N., Kirk P.M., Bourgoin T., DeWalt R.E., Decock W., Nieukerken E. van, Zarucchi J., Penev L., eds. (2019). Species 2000 & ITIS Catalogue of Life, 2019 Annual Checklist. Digital resource at www.catalogueoflife.org/annual-checklist/2019. Species 2000: Naturalis, Leiden, the Netherlands. ISSN 2405-884X. Accessed 21 July 2021.

**Velvet Worms (Udeonychophora)** — Oliveira, I.S., Hering, L. and Mayer, G. (2006-2022). The Onychophora Website. Digital resource at http://www.onychophora.com/index.htm. Accessed 23 June 2022. For number of described species see http://www.onychophora.com/list.htm.

**Horseshoe Crabs (Merostomata)** — Class Merestomata excludes the fossil sea scorpions; only four species are extant today: Roskov Y., Ower G., Orrell T., Nicolson D., Bailly N., Kirk P.M., Bourgoin T., DeWalt R.E., Decock W., Nieukerken E. van, Zarucchi J., Penev L., eds. (2019). Species 2000 & ITIS Catalogue of Life, 2019 Annual Checklist. Digital resource at www.catalogueoflife.org/annual-checklist/2019. Species 2000: Naturalis, Leiden, the Netherlands. ISSN 2405-884X. Accessed 04 March 2021.

**Others** – "Others" includes all of the invertebrate groups listed in Catalog of Life that are not included in the groups listed above. Roskov Y., Ower G., Orrell T., Nicolson D., Bailly N., Kirk P.M., Bourgoin T., DeWalt R.E., Decock W., Nieukerken E. van, Zarucchi J., Penev L., eds. (2019). Species 2000 & ITIS Catalogue of Life, 2019 Annual Checklist. Digital resource at www.catalogueoflife.org/annual-checklist/2019. Species 2000: Naturalis, Leiden, the Netherlands. ISSN 2405-884X. Accessed 24 November 2021).

### Plants

**Mosses** – Christenhusz, M.J.M. and Byng, J.W. 2016. The number of known plant species in the world and its annual increase. Phytotaxa. 261(3): 201-217. http://dx.doi.org/10.11646/phytotaxa.261.3.1

**Ferns and allies** – State of the World's Plants 2017: https://stateoftheworldsplants.org/2017/report/SOTWP_2017.pdf

**Gymnosperms** – Christenhusz, M.J.M. *et al* . (2011). A new classification and linear sequence of extant gymnosperms. Phytotaxa.19: 55–70 (cited in State of the World's Plants 2017: https://stateoftheworldsplants.org/2017/report/SOTWP_2017.pdf).

**Flowering Plants** (Magnoliophyta = Magnoliopsida+Liliopsida) – State of the World's Plants 2017: https://stateoftheworldsplants.org/2017/report/SOTWP_2017.pdf.

### Fungi & Protists

**Lichens** - The estimated total number of lichen species currently ranges between 17,000 (Chapman 2009) and 28,000 (Thell *et al* . 2012). The figure presented in Table 1a will be updated as soon as a more accurate figure can be confirmed.
Chapman, A.D. 2009 . Numbers of Living Species in Australia and the World, 2nd edition. Australian Biological Resources Study, Canberra.
Available at: http://www.environment.gov.au/biodiversity/abrs/publications/other/species-numbers/2009/04-04-groups-fungi.html#lichen.
Accessed 02 September 2010.
Thell, A., Crespo, A. Divakar, P.K., Käarnefelt, I., Leavitt, S.D., Lumbsch, H.T. and Seaward, M.R.D. 2012. A review of the lichen family Parmeliaceae - history, phylogeny and current taxonomy. *Nordic Journal of Botany* 30(6): 641-664

**Mushrooms, brackets, rusts, smuts, jelly fungi, etc.** - Ascomycota and Basidiomycota (excluding the lichenised species).
Kirk P.M. (2019). Species Fungorum (version Oct 2017). In: Species 2000 & ITIS Catalogue of Life, 2019 Annual Checklist (Roskov Y., Ower G., Orrell T., Nicolson D., Bailly N., Kirk P.M., Bourgoin T., DeWalt R.E., Decock W., Nieukerken E. van, Zarucchi J., Penev L., eds). Digital resource at www.catalogueoflife.org/annual-checklist/2019. Species 2000: Naturalis, Leiden, the Netherlands. ISSN 2405-884X. Accessed 24 November 2021

**Green (Charophyta, Chlorophyta), Red (Rhodophyta) and Brown (Ochrophyta) Algae** – From Guiry, M.D. and Guiry, G.M. 2022. AlgaeBase. World-wide electronic publication, National University of Ireland, Galway. http://www.algaebase.org. Accessed on 23 June 2022. For taxonomy search, see https://www.algaebase.org/browse/taxonomy/

**Table S5: Validation of the 3D models.** The Fish ALOX 3D structures were generated by homology modeling using Prime module of Schrodinger software. Crystal co-ordinates of rabbit ALOX15 (PDB ID: 2P0M, chain A) were used to construct the 3D models. The psi/phi Ramachandran plot was obtained from PROCHECK analysis (Laskowski et al., 1993) and the statistics of non-bonded atom-atom interactions in comparison with reliable high-resolution structures was obtained from ERRAT analysis (Colovos et al., 1993). **Expressed as the percentage of the protein for which the calculated error value falls below the 95% rejection limit. Good high-resolution structures generally produce values around 95% or higher. For lower resolutions (2.5 to 3A) the average overall quality factor is around 91%.

| | Program used | | | | |
|---|---|---|---|---|---|
| | ERRAT | PROCHECK (Ramachandran plot) | | | |
| Enzyme | Overall Quality Factor** | % of residues in most favoured regions | % of residues in additional allowed regions | % of residues in generously allowed regions | % of residues in disallowed regions |
| Rabbit ALOX15 (Template: 2P0M) | 89.9068 | 87.4 | 12.5 | 0.2 | 0.0 |
| *N. fuzeri* | 86.9301 | 84.8 | 13.0 | 1.5 | 0.7 |
| *P. nyererei* | 85.7812 | 83.0 | 14.5 | 1.7 | 0.8 |
| *S. formosus* | 85.3774 | 83.5 | 14.5 | 1.3 | 0.7 |
| *D. rerio* | 83.2278 | 82.2 | 15.3 | 1.5 | 1.0 |
| Human ALOX12 | 90.3226 | 86.0 | 12.3 | 1.2 | 0.5 |

**Table S6: Validation of the 3D models.** The Fish ALOX 3D structures were generated by homology modeling using Prime module of Schrodinger software. Crystal co-ordinates of human ALOX15B (PDB ID: 4NRE, chain A) were used to construct the 3D models. The psi/phi Ramachandran plot was obtained from PROCHECK analysis (Laskowski et al., 1993). and the statistics of non-bonded atom-atom interactions in comparison with reliable high-resolution structures was obtained from ERRAT analysis (Colovos et al., 1993). **Expressed as the percentage of the protein for which the calculated error value falls below the 95% rejection limit. Good high-resolution structures generally produce values around 95% or higher. For lower resolutions (2.5 to 3A) the average overall quality factor is around 91%.

| | Program used | | | | |
|---|---|---|---|---|---|
| | ERRAT | PROCHECK (Ramachandran plot) | | | |
| Enzyme | Overall Quality Factor** | % of residues in most favoured regions | % of residues in additional allowed regions | % of residues in generously allowed regions | % of residues in disallowed regions |
| Human ALOX15B (Template:4NRE) | 89.7898 | 90.4 | 9.6 | 0.0 | 0.0 |
| *N. fuzeri* | 88.9561 | 90.0 | 8.7 | 0.8 | 0.5 |
| *P. nyererei* | 89.5455 | 89.4 | 9.7 | 0.7 | 0.2 |
| *S. formosus* | 91.933 | 89.7 | 9.3 | 0.8 | 0.2 |
| *D. rerio* | 90.7716 | 88.6 | 10.1 | 1.0 | 0.3 |
| Human ALOX12 | 85.9112 | 88.7 | 10.1 | 0.9 | 0.3 |

References


1. Laskowski R A, MacArthur M W, Moss D S, Thornton J M (1993). PROCHECK - a program to check the stereochemical quality of protein structures. J. App. Cryst., 26, 283-291.

2. Colovos C, Yeates TO. Verification of protein structures: patterns of nonbonded atomic interactions. Protein Sci. 1993 Sep;2(9):1511-9. doi: 10.1002/pro.5560020916. PMID: 8401235; PMCID: PMC2142462.

**Figure S1A: Dual amino acid sequence alignment of human ALOX15 and the putative ALOX15 ortholog of *N. furzeri*.** Dual amino acid sequence alignments were carried out with the Emboss Needle (https://www.ebi.ac.uk/Tools/psa/emboss_needle/) program. The conserved iron ligands are indicated.

**A**

```
Homo15_NP_001      1 MGLYRIRVSTGASLYAGSNNQVQLWLVGQHGEAAL----GKRLWPARGKE     46
                     |.:|.:.|:||.|.|:|:||.:.:.|:|..||:..    ...|...||..
Notho15_XP_01      1 MEVYTVTVATGTSEYSGTNNYIFVTLIGDKGESERTLLDNPGLDFCRGAV     50

Homo15_NP_001     47 TELKVEVPEYLGPLLFVKLRKRHLLKDDAWFCNWISVQGPGAGDEVRFPC     96
                     .:.||.....||.||.|:|.|.....:|.|||.::.|:.|..|....|||
Notho15_XP_01     51 DQYKVSTQSRLGTLLLVRLEKEKYWVEDNWFCRYVMVEPPDGGKVRTFPC    100

Homo15_NP_001     97 YRWVEGNGVLSLPEGTGRTVGEDPQGLFQKHREEELEERRKLYRWGNWKD    146
                     |||:.||..:.:.|||.:|:.:|.......||:.||:||:|.|||..|..
Notho15_XP_01    101 YRWLIGNTKVEIREGTAKTLLDDSLPTVVAHRKAELQERQKTYRWVTWAK    150

Homo15_NP_001    147 GLILNMAGAKLYDLPVDERFLEDKRVDFEVSLAKGLADLAIKD-SLNVLT    195
                     |:...:......|||.|.||..:||.|||.||...|.:|::|. ::....
Notho15_XP_01    151 GIPRCIDAKTEADLPQDVRFDNEKRSDFEHSLHYALLELSLKKLAIRFGK    200

Homo15_NP_001    196 CWKDLDDFNRIFWCGQSKLAERVRDSWKEDALFGYQFLNGANPVVLRRSA    245
                     .|.|||||.||||..:|.:||...:.||||..||||.|||.||.:::|..
Notho15_XP_01    201 SWNDLDDFKRIFWKLRSPIAEYCMEHWKEDWFFGYQCLNGCNPRMIQRCQ    250

Homo15_NP_001    246 HLPARLVFPPGMEELQAQ------LEKELEGGTLFEADFSLLDGIKANVI    289
                     .||..  ||...:.:|:.      |.|||:.|.::..|:|::|||.||.|
Notho15_XP_01    251 KLPEN--FPVTADMVQSSMASKTTLNKELQAGNIYLLDYSIMDGIPANTI    298

Homo15_NP_001    290 LCSQQHLAAPLVMLKLQPDGKLLPMVIQLQLPRTGSP--PPPLFLPTDPP    337
                     ....|::|||:.:|...||..|:|:.|||:    .||  ..|:|||.|.|
Notho15_XP_01    299 KGKLQYIAAPICLLYQHPDDGLIPIAIQLE----QSPGLETPIFLPKDAP    344

Homo15_NP_001    338 MAWLLAKCWVRSSDFQLHELQSHLLRGHLMAEVIVVATMRCLPSIHPIFK    387
                     :|||.||.|||.|:||:.:|.|||||.||:.||..||||:|.||::|||:|
Notho15_XP_01    345 LAWLFAKMWVRHSEFQVFQLLSHLLRTHLVVEVFCVATLRQLPAVHPIYK    394

Homo15_NP_001    388 LIIPHLRYTLEINVRARTGLVSDMGIFDQIMSTGGGGHVQLLKQAGAFLT    437
                     |:.||||||||||.|.||.|:|..|||.:::||||.|.:.|.::....||
Notho15_XP_01    395 LLAPHLRYTLEINCRGRTQLISANGIFKRVVSTGGDGLLILAQREYKVLT    444

Homo15_NP_001    438 YSSFCPPDDLADRGLLGVKSSFYAQDALRLWEIIYRYVEGIVSLHYKTDV    487
                     |.|..|..|.:|||:..:.:.:.||.:.:|.|||.::.:|..:|:|:|.||.
Notho15_XP_01    445 YRSLQPHYDFSDRGVSQLPNYFYREHSLMLWEAVHSFVSSMVNLYYHTDQ    494

Homo15_NP_001    488 AVKDDPELQTWCREITEIGLQGAQDRGFPVSLQARDQVCHFVTMCIFTCT    537
                     .|:.|||||.|.|:|:..|.......|...||.:|::....:.:.|||.|
Notho15_XP_01    495 DVQKDPELQAWIRDISLEGFTELPSFGLASSLSSREELSTLLAVAIFTST    544

Homo15_NP_001    538 GQHASVHLGQLDWYSWVPNAPCTMRLPPPTTK-DATLETVMATLPNFHQA    586
                     .|||:.:.||.||.:||||.|||||||||..| |.|:|.:|||||:..|:
Notho15_XP_01    545 AQHAATNNGQFDWCAWVPNTPCTMRLPPPADKDDVTMERIMATLPDVSQS    594

Homo15_NP_001    587 SLQMSITWQLGRRQPVMVAVGQHEEEYFSGPEPKAVLKKFREELAALDKE    636
                     .:||:|||.|||.||..:.:||:.|::|:..|...|:..||.:|..::..
Notho15_XP_01    595 CVQMAITWHLGRAQPDAIPLGQYTEDHFTEEEALEVVDSFRTKLKEIENY    644

Homo15_NP_001    637 IEIRNAKLDMPYEYLRPSVVENSVAI    662
                     |..:||.||:.|.:|.||.||||:.|
Notho15_XP_01    645 ILDQNAGLDLQYLFLLPSRVENSITI    670
```

**Figure S1B: Dual amino acid sequence alignment of human ALOX15 and the putative ALOX15 ortholog of *P. nyererei*.** Dual amino acid sequence alignments were carried out with the Emboss Needle (https://www.ebi.ac.uk/Tools/psa/emboss_needle/) program. The conserved iron ligands are indicated.

**B**

```
Homo15_NP_001      1 MGLYRIRVSTGASLYAGSNNQVQLWLVGQHGEAALGKRLWP----ARGKE     46
                     |.:|.:.|:||.|.|:|:||.:.|.|||::||:...:...|    .||..
Punda15_XP_00      1 MEVYTVTVATGTSEYSGTNNYIYLTLVGENGESERTQLDNPGLDFCRGAV     50

Homo15_NP_001     47 TELKVEVPEYLGPLLFVKLRKRHLLKDDAWFCNWISVQGPGAGDEVRFPC     96
                     .:.||..|..||.:|.|:|.|:....:|.|||.:::|:.|..|..:.|||
Punda15_XP_00     51 DQYKVTSPSPLGSVLLVRLEKQRYWVEDNWFCQYVTVEPPDGGKVLTFPC    100

Homo15_NP_001     97 YRWVEGNGVLSLPEGTGRTVGEDPQGLFQKHREEELEERRKLYRWGNWKD    146
                     |||..|:..:.:.|||.:|:..|......:||..||:||:|.|||..|..
Punda15_XP_00    101 YRWFVGDVKMEIREGTAKTLTGDSALQLLEHRRTELQERQKTYRWVTWAP    150

Homo15_NP_001    147 GLILNMAGAKLYDLPVDERFLEDKRVDFEVSLAKGLADLAIKDSLNVLT-    195
                     |:...:......|||.|.||..:||.|||.||...|.:|::|.     ||
Punda15_XP_00    151 GIPRCVDAETEADLPQDARFDNEKRSDFEHSLHYALLELSLKK----LTI    196

Homo15_NP_001    196 ----CWKDLDDFNRIFWCGQSKLAERVRDSWKEDALFGYQFLNGANPVVL    241
                         .|.||:||.||||..:|.:||...:.||||..||||.|||:||.::
Punda15_XP_00    197 WFGKSWDDLEDFKRIFWKLKSPIAEYCMNHWKEDWFFGYQCLNGSNPRMI    246

Homo15_NP_001    242 RRSAHLPARLVFPPGMEEL----QAQLEKELEGGTLFEADFSLLDGIKAN    287
                     :|...||..|.....|.:.    :..|.|||:.|.::..|::::|||.:|
Punda15_XP_00    247 QRCKKLPENLPVTADMVQRSMAGRTNLNKELKAGNIYLLDYAIMDGIPSN    296

Homo15_NP_001    288 VILCSQQHLAAPLVMLKLQPDGKLLPMVIQLQLPRTGSPPPPLFLPTDPP    337
                     .|....|::|||:.:|....||..|:|:.|||:  :|.....|:|||:|||
Punda15_XP_00    297 TIKGHPQYIAAPICLLYQHPDEGLIPIAIQLE--QTPGRDTPIFLPSDPP    344

Homo15_NP_001    338 MAWLLAKCWVRSSDFQLHELQSHLLRGHLMAEVIVVATMRCLPSIHPIFK    387
                     :||||||.|||.|:||:.:|.|||||.||:.||..|||:|.||::|||:|
Punda15_XP_00    345 LAWLLAKMWVRHSEFQVFQLLSHLLRTHLVVEVFCVATLRQLPAVHPIYK    394

Homo15_NP_001    388 LIIPHLRYTLEINVRARTGLVSDMGIFDQIMSTGGGGHVQLLKQAGAFLT    437
                     |:.||||||||||.|.||.|:|..|||.:::||||.|.:.|.::....||
Punda15_XP_00    395 LLAPHLRYTLEINCRGRTQLISANGIFKRVVSTGGDGLLILAQREYKVLT    444

Homo15_NP_001    438 YSSFCPPDDLADRGLLGVKSSFYAQDALRLWEIIYRYVEGIVSLHYKTDV    487
                     |.|..|..|..|||:..:.:.||...:|.|||.|:.:|..:|:|:|::|.
Punda15_XP_00    445 YRSIQPLYDFCDRGVSQLPNYFYKDHSLMLWEAIHSFVSSMVNLYYQSDH    494

Homo15_NP_001    488 AVKDDPELQTWCREITEIGLQGAQDRGFPVSLQARDQVCHFVTMCIFTCT    537
                     .|::|.|||.|.|:|||.|.....:.|.|..|.:|:::|..:.:.|||.|
Punda15_XP_00    495 DVQEDLELQAWIRDITEEGFTELPNFGLPSKLSSREELCTLLAVAIFTST    544

Homo15_NP_001    538 GQHASVHLGQLDWYSWVPNAPCTMRLPPPTTKDA-TLETVMATLPNFHQA    586
                     .|||:.:.||.||.:||||.|||||.||||.||| |::.:|||||:..|:
Punda15_XP_00    545 AQHAATNNGQFDWCAWVPNTPCTMRQPPPTDKDAVTMDMIMATLPDVSQS    594

Homo15_NP_001    587 SLQMSITWQLGRRQPVMVAVGQHEEEYFSGPEPKAVLKKFREELAALDKE    636
                     .:||:|||.|||.||..:.:|::.|::|:..:...|:.:||.||..::|.
Punda15_XP_00    595 CVQMAITWHLGRAQPDAIPLGRYTEDHFTEAKALEVIDRFRVELKEIEKH    644

Homo15_NP_001    637 IEIRNAKLDMPYEYLRPSVVENSVAI    662
                     |...|..|::.|.:|.||.:|||:.|
Punda15_XP_00    645 ILTENEGLELQYLFLLPSRIENSITI    670
```

**Figure S1C: Dual amino acid sequence alignment of human ALOX15 and the putative ALOX15 ortholog of *S. formosus*** Dual amino acid sequence alignments were carried out with the Emboss Needle (https://www.ebi.ac.uk/Tools/psa/emboss_needle/) program. The conserved iron ligands are indicated.

**C**

```
Homo15_NP_001      1 MGLYRIRVSTGASLYAGSNNQVQLWLVGQHGEAAL------GKRLWPARG     44
                     |. |.:.|:||.|.|:|:||.|.:.:||:.||:..      |..|  .||
Sclero15_XP_0      1 MD-YTVTVATGTSEYSGTNNYVYVTVVGEKGESERTVLDNPGLDL--CRG     47

Homo15_NP_001     45 KETELKVEVPEYLGPLLFVKLRKRHLLKDDAWFCNWISVQGPGAGDEVRF     94
                     ...:.:|.....||.||.|:|.|.....:|.|||.:::|..|. ||.:.|
Sclero15_XP_0     48 AVDKYRVHSAASLGQLLLVRLEKERYWVEDNWFCRYVAVTSPD-GDTLTF     96

Homo15_NP_001     95 PCYRWVEGNGVLSLPEGTGRTVGEDPQGLFQKHREEELEERRKLYRWGNW    144
                     |||||:.||..:.|.|||.:.:.:|...|..:||..||:||:|:|||..|
Sclero15_XP_0     97 PCYRWLVGNVKVELREGTAKKLNDDTLSLLLEHRRAELQERQKIYRWQAW    146

Homo15_NP_001    145 KDGLILNMAGAKLYDLPVDERFLEDKRVDFEVSLAKGLADLAIKD-SLNV    193
                     ..|:...:......|||.|.||..:||.|||.||...|.:|::|. :|..
Sclero15_XP_0    147 APGIPKCIDAKSEADLPQDARFDNEKRSDFEGSLQFALLELSLKTLALKF    196

Homo15_NP_001    194 LTCWKDLDDFNRIFWCGQSKLAERVRDSWKEDALFGYQFLNGANPVVLRR    243
                     ...|..||||.||||..:|.:||.|..:|:||.|||||||||.||.::::
Sclero15_XP_0    197 GKSWDSLDDFKRIFWKLRSPVAEYVMKNWREDWLFGYQFLNGCNPRMIQQ    246

Homo15_NP_001    244 SAHLPARLVFPPGMEELQAQ------LEKELEGGTLFEADFSLLDGIKAN    287
                     ...||..  ||...:.:|..      |.||::.|.::..:::::|||.||
Sclero15_XP_0    247 CRKLPDN--FPVSEDMVQGSMGPKMTLSKEMKAGNIYLVNYAIMDGIPAN    294

Homo15_NP_001    288 VILCSQQHLAAPLVMLKLQPDGKLLPMVIQLQLPRTGSPPPPLFLPTDPP    337
                     ||....|||||||.:|...|:..|:|:.|||.  :...|..|:|||:|||
Sclero15_XP_0    295 VIRNRPQHLAAPLCLLYEHPESGLIPIAIQLD--QNPGPDTPIFLPSDPP    342

Homo15_NP_001    338 MAWLLAKCWVRSSDFQLHELQSHLLRGHLMAEVIVVATMRCLPSIHPIFK    387
                     :||||||.|||.|:||:.::.|||||.||:.||..|||:|.||::|||:|
Sclero15_XP_0    343 LAWLLAKIWVRHSEFQVFQVLSHLLRTHLVIEVFCVATLRQLPAVHPIYK    392

Homo15_NP_001    388 LIIPHLRYTLEINVRARTGLVSDMGIFDQIMSTGGGGHVQLLKQAGAFLT    437
                     |:.||||||||||.|.||.|:|..|||.:::||||.|.:.|.::....||
Sclero15_XP_0    393 LLTPHLRYTLEINCRGRTQLISSNGIFKRVVSTGGEGLLVLAQKEYKVLT    442

Homo15_NP_001    438 YSSFCPPDDLADRGLLGVKSSFYAQDALRLWEIIYRYVEGIVSLHYKTDV    487
                     |.|..|..|..:||:..:|..||.:|::.||:.|:.:..|:|.|:|.:|.
Sclero15_XP_0    443 YRSLQPSVDFHERGVTKLKGYFYKEDSMMLWDAIHSFTWGMVCLYYSSDT    492

Homo15_NP_001    488 AVKDDPELQTWCREITEIGLQGAQDRGFPVSLQARDQVCHFVTMCIFTCT    537
                     .|.:|.|||.|.:||...|.......|....|:.|:::...:::.|||.|
Sclero15_XP_0    493 DVAEDEELQAWIQEIANEGFVDVPKFGLSSELKTREELNTLLSVVIFTST    542

Homo15_NP_001    538 GQHASVHLGQLDWYSWVPNAPCTMRLPPPTTKDA-TLETVMATLPNFHQA    586
                     .|||:.:.||.||.:|:||.|||||.||||.||| |:|.|:.|||:..|:
Sclero15_XP_0    543 AQHAATNNGQFDWCAWIPNTPCTMRCPPPTDKDAITMELVIDTLPDVSQS    592

Homo15_NP_001    587 SLQMSITWQLGRRQPVMVAVGQHEEEYFSGPEPKAVLKKFREELAALDKE    636
                     .:||:|||.|||.||..:.:||:.||||:.|..|.::::||:||..:::.
Sclero15_XP_0    593 CMQMAITWHLGRLQPDAIQLGQYTEEYFTEPRAKELIERFRQELRDVEEH    642

Homo15_NP_001    637 IEIRNAKLDMPYEYLRPSVVENSVAI    662
                     |..:|..|::||.||.||.:|||:.|
Sclero15_XP_0    643 IAKKNEGLELPYLYLCPSRIENSITI    668
```

**Figure S1D: Dual amino acid sequence alignment of human ALOX15 and the putative ALOX15 ortholog of *H. burtoni*.** Dual amino acid sequence alignments were carried out with the Emboss Needle (https://www.ebi.ac.uk/Tools/psa/emboss_needle/) program. The conserved iron ligands are indicated.

**D**

```
Homo15_NP_001      1 MGLYRIRVSTGASLYAGSNNQVQLWLVGQHGEAALGKRLWP----ARGKE     46
                     |.:|.:.|:||.|.|:|:||.:.|.|||::||:...:...|    .||..
Haplo15_XP_00      1 MEVYTVTVATGTSEYSGTNNYIYLTLVGENGESERTQLDNPGLDFCRGAV     50

Homo15_NP_001     47 TELKVEVPEYLGPLLFVKLRKRHLLKDDAWFCNWISVQGPGAGDEVRFPC     96
                     .:.||..|..||.:|.|:|.|:....:|.|||.:::|:.|..|..:.|||
Haplo15_XP_00     51 DQYKVTSPSPLGSVLLVRLEKQRYWVEDNWFCQYVTVEPPDGGKVLTFPC    100

Homo15_NP_001     97 YRWVEGNGVLSLPEGTGRTVGEDPQGLFQKHREEELEERRKLYRWGNWKD    146
                     |||..|:..:.:.|||.:|:..|......:||..||:||:|.|||..|..
Haplo15_XP_00    101 YRWFVGDVKMEIREGTAKTLTGDSALQLLEHRRTELQERQKTYRWVTWAP    150

Homo15_NP_001    147 GLILNMAGAKLYDLPVDERFLEDKRVDFEVSLAKGLADLAIKDSLNVLT-    195
                     |:...:......|||.|.||..:||.|||.||...|.:|::|.    ||
Haplo15_XP_00    151 GIPRCVDAETEADLPQDARFDNEKRSDFEHSLHYALLELSLKK----LTI    196

Homo15_NP_001    196 ----CWKDLDDFNRIFWCGQSKLAERVRDSWKEDALFGYQFLNGANPVVL    241
                         .|.||:||.||||..:|.:||...:.||||..||||.|||:||.::
Haplo15_XP_00    197 WFGKSWDDLEDFKRIFWKLKSPIAEYCMNHWKEDWFFGYQCLNGSNPRMI    246

Homo15_NP_001    242 RRSAHLPARLVFPPGMEEL----QAQLEKELEGGTLFEADFSLLDGIKAN    287
                     :|...||..|.....|.:.    :..|.|||:.|.::..|::::|||.:|
Haplo15_XP_00    247 QRCKKLPENLPVTADMVQRSMAGRTNLNKELKAGNIYLLDYAIMDGIPSN    296

Homo15_NP_001    288 VILCSQQHLAAPLVMLKLQPDGKLLPMVIQLQLPRTGSPPPPLFLPTDPP    337
                     .|....|::|||:.:|...||..|:|:.|||:  :|.....|:|||:|||
Haplo15_XP_00    297 TIKGHPQYIAAPICLLYQHPDEGLIPIAIQLE--QTPGRDTPIFLPSDPP    344

Homo15_NP_001    338 MAWLLAKCWVRSSDFQLHELQSHLLRGHLMAEVIVVATMRCLPSIHPIFK    387
                     :||||||.|||.|:||:.:|.|||||.||:.||..|||:|.||::|||:|
Haplo15_XP_00    345 LAWLLAKMWVRHSEFQVFQLLSHLLRTHLVVEVFCVATLRQLPAVHPIYK    394

Homo15_NP_001    388 LIIPHLRYTLEINVRARTGLVSDMGIFDQIMSTGGGGHVQLLKQAGAFLT    437
                     |:.||||||||||.|.||.|:|..|||.:::||||.|.:.|.:::....||
Haplo15_XP_00    395 LLAPHLRYTLEINCRGRTQLISANGIFKRVVSTGGDGLLILAQREYKVLT    444

Homo15_NP_001    438 YSSFCPPDDLADRGLLGVKSSFYAQDALRLWEIIYRYVEGIVSLHYKTDV    487
                     |.|..|..|..|||:..:.:.||...:|.|||.|:..:|.:.|:  ...:
Haplo15_XP_00    445 YRSIQPLYDFCDRGVSQLPNYFYKDHSLMLWEAIHSQLEFLASI--LNPL    492

Homo15_NP_001    488 AVKDDPELQTW--CREITEIG------------LQGAQDRGFPVSLQARD    523
                     ....:..:|..  |.|..:..            |.|:...|.|..|.:|:
Haplo15_XP_00    493 VGSSEHHIQNTLDCVEKQKFSAGYPVSVYPVYPLNGSCVPGLPSKLSSRE    542

Homo15_NP_001    524 QVCHFVTMCIFTCTGQHASVHLGQLDWYSWVPNAPCTMRLPPPTTKDA-T    572
                     ::|..:.:.|||.|.|||:.:.||.||.:||||.|||||.||||.||| |
Haplo15_XP_00    543 ELCTLLAVAIFTSTAQHAATNNGQFDWCAWVPNTPCTMRQPPPTDKDAVT    592

Homo15_NP_001    573 LETVMATLPNFHQASLQMSITWQLGRRQPVMVAVGQHEEEYFSGPEPKAV    622
                     ::.:|||||:..|:.:|:|||.|||.||..:.:|::.|::|:.:...|
Haplo15_XP_00    593 MDMIMATLPDVSQSCVQMAITWHLGRAQPDAIPLGRYTEDHFTEAKALEV    642

Homo15_NP_001    623 LKKFREELAALDKEIEIRNAKLDMPYEYLRPSVVENSVAI    662
                     :.:||.||..::|.|...|..|::.|.:|.||.:|||:.|
Haplo15_XP_00    643 IDRFRVELKEIEKHILTENEGLELQYLFLLPSRIENSITI    682
```

**Figure S1E: Dual amino acid sequence alignment of human ALOX15 and the putative ALOX15 ortholog of *T. rubripes*.** Dual amino acid sequence alignments were carried out with the Emboss Needle (https://www.ebi.ac.uk/Tools/psa/emboss_needle/) program. The conserved iron ligands are indicated.

# E

```
Homo15_NP_001      1 MGLYRIRVSTGASLYAGSNNQVQLWLVGQHGEAAL----GKRLWPARGKE     46
                     |..|.:.|:||.|.|:|:||.:.:.|:|:|||:..    ...|...||..
Taki15_XP_003      1 METYTVTVATGTSEYSGTNNYIFVTLLGEHGESERTLLDNPGLDFCRGAV     50

Homo15_NP_001     47 TELKVEVPEYLGPLLFVKLRKRHLLKDDAWFCNWISVQGPGAGDEVRFPC     96
                     .:.||..|..||.:..|:|.|:....:|.|||.::.|:.||...|:.|||
Taki15_XP_003     51 DKYKVISPCPLGSIFMVRLEKQKYWVEDNWFCRYVKVEPPGGRSELTFPC    100

Homo15_NP_001     97 YRWVEGNGVLSLPEGTGRTVGEDPQGLFQKHREEELEERRKLYRWGNWKD    146
                     |||:.|:..:.:.|||.:.:..| ..|.|.||:|||:||:|.|||.:|..
Taki15_XP_003    101 YRWLIGDVKVEIREGTAKILYND-NNLLQAHRKEELKERQKKYRWVSWTP    149

Homo15_NP_001    147 GLILNMAGAKLYDLPVDERFLEDKRVDFEVSLAKGLADLAIKD-SLNVLT    195
                     |:...:......||..|.||..:|:.||:.||...|.:|.:|. ::....
Taki15_XP_003    150 GIPRCIDAETEADLHPDVRFDNEKKSDFQYSLQYALLELCLKKFAMMFGK    199

Homo15_NP_001    196 CWKDLDDFNRIFWCGQSKLAERVRDSWKEDALFGYQFLNGANPVVLRRSA    245
                     .|.:|:||..:||..:|.|||...|.||||:.||||.:||:||.::||..
Taki15_XP_003    200 SWNNLEDFKCLFWNLRSPLAEYCMDHWKEDSFFGYQCMNGSNPRMIRRCQ    249

Homo15_NP_001    246 HLPARLVFPPGMEELQA------QLEKELEGGTLFEADFSLLDGIKANVI    289
                     .||.:  ||.....:|:      .|..||:.|.::..|:::.|||.:|.|
Taki15_XP_003    250 QLPGK--FPVTSNMVQSSMNSRTDLANELKAGNIYILDYAITDGIPSNTI    297

Homo15_NP_001    290 LCSQQHLAAPLVMLKLQPDGKLLPMVIQLQLPRTGSPP---PPLFLPTDP    336
                     ....|::|||:.:|...||..|.|:.|||     |..|   .|:||||||
Taki15_XP_003    298 KGVPQYIAAPICLLYNHPDDGLKPIAIQL-----GQTPGVDTPIFLPTDP    342

Homo15_NP_001    337 PMAWLLAKCWVRSSDFQLHELQSHLLRGHLMAEVIVVATMRCLPSIHPIF    386
                     |:||||||.|||:::||:.:|.|||||.||:.||..|||:|.||.:||::
Taki15_XP_003    343 PLAWLLAKMWVRNTEFQVFQLLSHLLRTHLVMEVFCVATLRQLPDVHPVY    392

Homo15_NP_001    387 KLIIPHLRYTLEINVRARTGLVSDMGIFDQIMSTGGGGHVQLLKQAGAFL    436
                     |
Taki15_XP_003    393 K-------------------------------------------------    393

Homo15_NP_001    437 TYSSFCPPDDLADRGLLGVKSSFYAQDALRLWEIIYRYVEGIVSLHYKTD    486
                     ||.|..|.:|.||||:..:...||.:.:|.:|:.|.::|.|:|||:|::|
Taki15_XP_003    394 TYRSLQPRNDFADRGVARLPKYFYLEHSLMVWDAIDKFVSGVVSLYYQSD    443

Homo15_NP_001    487 VAVKDDPELQTWCREITEIGLQGAQDRGFPVSLQARDQVCHFVTMCIFTC    536
                     ..|:.|||||.|.|:||:.|.......|.|..|.:|:::|..:::.|||.
Taki15_XP_003    444 QDVQQDPELQAWIRDITQEGFTELPSFGLPSKLCSREELCTLLSVAIFTA    493

Homo15_NP_001    537 TGQHASVHLGQLDWYSWVPNAPCTMRLPPPTTKDA-TLETVMATLPNFHQ    585
                     :.|||:.:.||.||.:|:||.|||||.|||..||| |:||:|||||:..|
Taki15_XP_003    494 SVQHAATNNGQFDWCAWIPNTPCTMRQPPPGDKDAVTMETIMATLPDISQ    543

Homo15_NP_001    586 ASLQMSITWQLGRRQPVMVAVGQHEEEYFSGPEPKAVLKKFREELAALDK    635
                     :.:||:|||.|||.||..|.:|.:.||||:..:...::.||:|||..:::
Taki15_XP_003    544 SCMQMAITWHLGRPQPDAVPLGHYSEEYFTETKTLELIDKFKEELREIEE    593

Homo15_NP_001    636 EIEIRNAKLDMPYEYLRPSVVENSVAI     662
                     :|..:|...::.|.:|.||.:|||:.|
Taki15_XP_003    594 QILQQNEGTELSYLFLLPSRIENSITI     620
```

**Figure S1F: Dual amino acid sequence alignment of human ALOX15 and the putative ALOX15 ortholog of *H. comes*.** Dual amino acid sequence alignments were carried out with the Emboss Needle (https://www.ebi.ac.uk/Tools/psa/emboss_needle/) program. The conserved iron ligands are indicated.

**F**

```
Homo15_NP_001      1 MGLYRIRVSTGASLYAGSNNQVQLWLVGQHGEAALGKRLWPARGKETELK     50

Hippo15_XP_01      1 --------------------------------------------------      0

Homo15_NP_001     51 VEVPEYLGPLLFVKLRKRHLLKDDAWFCNWISVQGPGAGDEVRFPCYRW-     99
                                                                 ...:|
Hippo15_XP_01      1 --------------------------------------------MSRQWR      6

Homo15_NP_001    100 ---VEGNGVLSLPEGT---------------GRTVGEDPQGLFQKHREEE    131
                        |...|.| :|.||               .:....|...|.|.||..:
Hippo15_XP_01      7 ATQVVFTGYL-VPAGTMLVTPGQHLPIFFSLAKIQSHDSSALLQAHRGTD     55

Homo15_NP_001    132 LEERRKLYRWGNWKDGLIILNMAGAKLYDLPVDERFLEDKRVDFEVSLAKG    181
                     ||:|:.:|||..|..|::..:......||..|.||...||.|||.:|...
Hippo15_XP_01     56 LEDRQAIYRWLTWALGILRYIYAQTENDLLQDVRFDNGKRSDFEHALHSA    105

Homo15_NP_001    182 LADLAIKDSLNVLTCWKDLDDFNRIFWCGQSKLAERVRD--SWKEDALFG    229
                     ..:|..|.::|....|   ||..||.|..||.|.:   |  .||||..||
Hippo15_XP_01    106 CXELLKKLAINFGKTW---DDLERISWELQSPLHD---DWMQWKEDCFFG    149

Homo15_NP_001    230 YQFLNGANPVVLR----RSAHLP-----ARLVFPPGMEELQAQLEKELEG    270
                     |..|||.||.::|    ...:.|     |:..||.|     :..:||.:.
Hippo15_XP_01    150 YGCLNGFNPRMIRTCKTTEGNFPVTAFTAQNSFPKG-----SNRDKEPQV    194

Homo15_NP_001    271 GTLFEADFSLLDGIKANVILCSQQHLAAPLVMLKLQPDGKLLPMVIQLQL    320
                     |.:|..:.:::|.|..|.|....|::..||.:|...|||..||:|
Hippo15_XP_01    195 GNVFSLEDTIMDWIPTNAIKGQLQNITGPLCLLNQHPDGVPLPVV-----    239

Homo15_NP_001    321 PRTGSPPPPLFLPTDPPMAWLLAKCWVRSSDFQLHELQSHLLRGHLMAEV    370
                     .:||                         |.||        :..|::.||
Hippo15_XP_01    240 NKTG-------------------------SKFQ--------VLSHMLVEV    256

Homo15_NP_001    371 IVVATMRCLPSIHPIFKLIIPHLRYTLEINVRARTGLVSDMGIFDQI---    417
                     ..|||:: .|||||:::      |||..||.|..|.|:|..|||.::
Hippo15_XP_01    257 FYVATLK-XPSIHPVYE------RYTQXINCRGHTRLISADGIFKRVSPY    299

Homo15_NP_001    418 ------MSTGGGGHVQLLKQAGAFLTYSSFCPPDDLADRGLLGVKSSFYA    461
                           :|||..|.:.|..:..:.|||.|.....|..|||:..:...||.
Hippo15_XP_01    300 LRDVCRVSTGAEGLLILAXRKXSDLTYRSLHHYLDFIDRGVSQLPKYFYQ    349

Homo15_NP_001    462 --------QDALRLW-EIIYRYVEGIVSLHYKTDVAVKDDPELQTWCREI    502
                             .|..|.. .::..:|.|::||:|.:|..|..|.|||.|.|::
Hippo15_XP_01    350 AXCCEKPYTDTQRAHSNVLISFVSGMMSLYYXSDKDVHQDAELQPWIRDV    399

Homo15_NP_001    503 TEIGLQGAQDRGFPVSLQARDQVCHFVTMCIFTCTGQHASVHLGQLDWYS    552
                     |:.||....|.|     |....|.|         |..:........||
Hippo15_XP_01    400 TQEGLAELPDFG-----QLSTSVTH---------THPYTITRRCSWDW--    433

Homo15_NP_001    553 WVPNAPCTMRLPPPTTKDATLETVMATLPNFHQASLQMSITWQLGRRQPV    602

Hippo15_XP_01    434 --------------------------------------------------    433

Homo15_NP_001    603 MVAVGQHEEEYFSGPEPKAVLKKFREELAALDKEIEIRNAKLDMPYEYLR    652

Hippo15_XP_01    434 --------------------------------------------------    433

Homo15_NP_001    653 PSVVENSVAI     662

Hippo15_XP_01    434 ----------     433
```

**Figure S2. LC-MS/MS analysis of the major oxygenation product formed from AA by the putative ALOX15 ortholog of *N. furzeri*.** The 12S-HETE peak prepared by NP/CP-HPLC from the AA oxygenation products of *N. furzeri* was analyzed by LC-MS/MS recording the relative abundance at m/z 319. Under these chromatographic conditions (see Materials and Methods) the product co-eluted with an authentic standard of 12S-HETE. The mass spectrum (left inset) was characterized by dominant mass ions at m/z 319 ($M^+$), m/z 301 ($M^+$-water) and 257 ($M^+$-water-carbon dioxide). The position of the OH group at the carbon backbone of AA is indicated by the dominant mass ion at m/z 179 (see right inset) and by the ion at m/t 135 (m/z 179 – carbon dioxide).

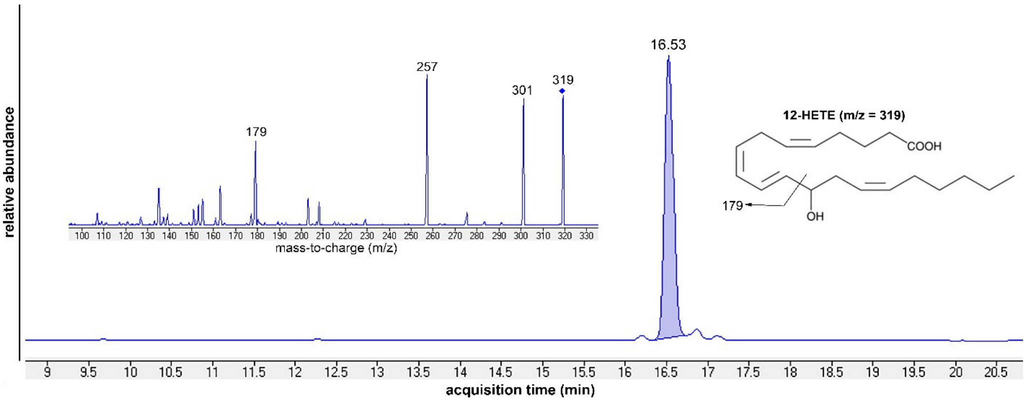


**Figure S3. Preferential oxygenation of AA and EPA when an equimolar mixture of AA, EPA and DHA was offered.** The putative bony fish ALOX15 orthologs (cellular lysis supernatants) were incubated in PBS with an equimolar (17 µM of each fatty acid) mixture of AA, EPA and DHA. After a 5 min incubation period the reaction products were reduced (addition of borohydride) and quantified by RP-HPLC. A) *N. furzeri*, B) *P. nyererei*, C) *S. formosus*. Four different measurements were carried out for each enzyme. The means of the relative catalytic activities of EPA oxygenation was set 100% and the relative oxygenase activities for the other fatty acids were calculated. Statistics: **A)** AA *vs.* EPA: unpaired U-test**. B) For all comparisons** unpaired t-test**. C)** AA *vs.* EPA: unpaired U-test, * p = ≤ 0.05, *** p = ≤ 0.001, **** p = ≤ 0.0001.

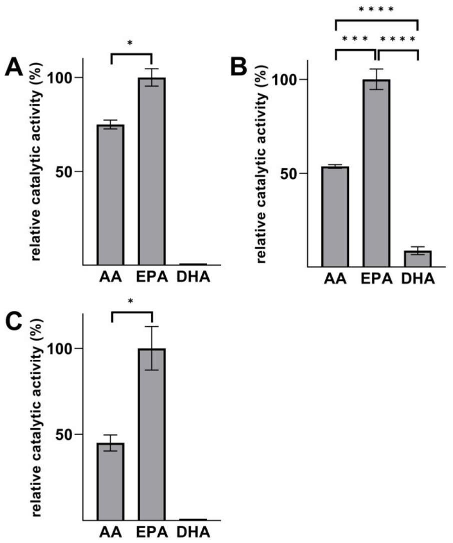

**Figure S4. The Ile418+Met419 motif of human ALOX15 is replaced by a Val+Val motif in the putative bony fish ALOX15 orthologs.** Multiple amino acid sequence alignments of the putative bony fish ALOX15 orthologs and the *D. rerio* LOX1 was carried out with the Emboss Needle (https://www.ebi.ac.uk/Tools/psa/emboss_needle/) program. The conserved Val+Val motif is indicated in yellow.

```
Danio15_NP_955912.1          IFKRVVSTGGEGLLILAQREYKVLTYRSLQPKFDFLDRGVTKVKGYVYRDYSLMLWDVIQ          479
Sclero15_XP_018588735.1      IFKRVVSTGGEGLLVLAQKEYKVLTYRSLQPSVDFHERGVTKLKGYFYKEDSMMLWDAIH          477
Notho15_XP_015813570.1       IFKRVVSTGGDGLLILAQREYKVLTYRSLQPHYDFSDRGVSQLPNYFYREHSLMLWEAVH          479
Punda15_XP_005753048.1       IFKRVVSTGGDGLLILAQREYKVLTYRSIQPLYDFCDRGVSQLPNYFYKDHSLMLWEAIH          479
```

**Figure S5. The reaction specificity of the putative bony fish ALOX15 orthologs was hardly changed when the Val+Val motif (Sloane determinants, see Figure S4) ) was mutated to Ile+Met.** The Val+Val to Ile+Met double mutants of the putative bony fish ALOX15 orthologs were prepared, the enzymes were incubated with AA and the patterns of the oxygenation products were analyzed by RP-HPLC. A) no enzyme control, B) *N. furzeri*, C) *P. nyererei*, *S. formosus*.

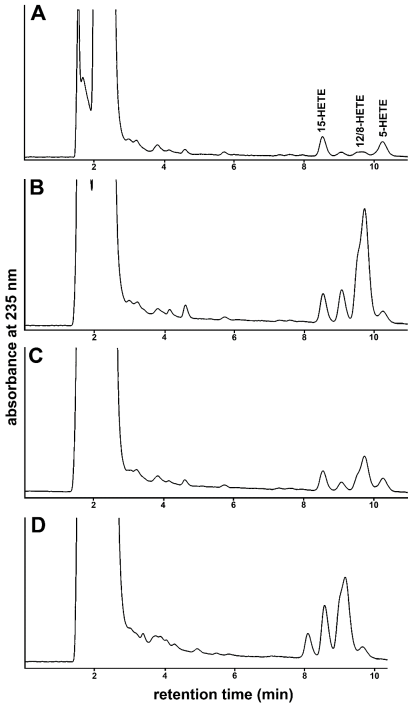


**Figure S6. *D. rerio* LOX1 and the putative ALOX15 orthologs of other bony fish carry a Gly at the Coffa determinant.** Multiple amino acid sequence alignments of the putative bony fish ALOX15 orthologs and the *D. rerio* LOX1 was carried out with the Emboss Needle (https://www.ebi.ac.uk/Tools/psa/emboss_needle/) program. The Coffa determinant (G), which immediately trails a highly conserved R (green), is indicated blue. Both amino acids are conserved in all bony fish enzymes.

```
Danio15 NP 955912.1          QVFQLLSHLLRTHLMVEVICVATLRQLPAVHPIYKLLTPHLRYTLEINCRGRTQLLSPEG          419
Sclero15 XP 018588735.1      QVFQVLSHLLRTHLVIEVFCVATLRQLPAVHPIYKLLTPHLRYTLEINCRGRTQLISSNG          417
Notho15_XP_015813570.1       QVFQLLSHLLRTHLVVEVFCVATLRQLPAVHPIYKLLAPHLRYTLEINCRGRTQLISANG          419
Punda15 XP 005753048.1       QVFQLLSHLLRTHLVVEVFCVATLRQLPAVHPIYKLLAPHLRYTLEINCRGRTQLISANG          419
```
